# Suicidal Pin Wounds

**Published:** 1888-04

**Authors:** 


### SUICIDAL PIN WOUNDS.

Mr. William Thompson reported to the Irish Academy a case of suicidal wound of the heart with a pin. The head of a pin was discovered in the fifth intercostal space, two inches and a half from the nipple—downwards and inwards. The pin had traversed the pericardium, and wounded the anterior wall of the left ventricle. The pericardium contained seventeen ounces and a half of bloody fluid, and there was a small rent in the wall a quarter of an inch in diameter, which was filled by blood-clot. The surface of the ventricle in contact with the pin was torn to the extent of nearly an inch ; a small vein was also wounded.—Dr. Foot referred to the case of Admiral Villeneuve, who commanded the French fleet at Trafalgar, and who some time afterwards pierced his heart with a long needle. A case came under his (Dr. Foot's) notice of a child into whose heart a needle went from its mother's dress as she was clasping it to her breast. She immediately ran with the child to the

Meath Hospital. He distinctly saw the oscillations of the needle caused by the action of the heart, and drew it out with a rotatory motion, in order to lessen the chance of hæmorrhage. The woman brought the child to him next day, nothing further happened. In Warsaw the latest treatment for cholera was puncturing the heart with a needle to stir up vitality.—Mr. Foy mentioned a case of a nobleman of Turin—a courtier of King Amadeo—who was killed by his wife, who drove a golden needle into his chest at a spot ascertained from anatomical plates to be over the heart. Fischer collected 453 cases of wounds of this sort, in the majority of which death resulted from the pericardium becoming filled with blood. In a case by Dr. Moxon, exhibited before the Clinical Society of London, a large pin was seen sticking in the chest of the patient, and moving with each motion of the heart, but apparently causing no trouble whatever to the patient. In another case a knitting-needle, shot from a toy pistol, perforated the right ventricle of a boy's heart, went into the auricle, and transfixed the valves, and yet the boy lived a considerable time. In another case a boy lived for five weeks after his heart had been pierced by a wooden peg three inches long. In the case of the woman who drove the thirty needles into herself, death resulted from a wound inflicted by only one of them upon the superior vena cava. In another case a pin found its way into the thoracic duct, and the patient bled to death. A most remarkable case was that of a medical student, who, while on a "spree," passed some pins into his heart. His pericardium was opened and the head of the pin was found outside the wall of the right ventricle. It was grasped, and a slight incision was made in the ventricle to facilitate its removal: but the systolic action of the heart carried it in, and the pin was still in the young man's ventricle, and occasioned him no trouble. Professor Christison recorded a case of a man wounded by a bullet, which found its way into the right ventricle of his heart, and it remained there for two years, occasioning no trouble, and he eventually died of pleurisy.—Dr. Finny said these cases showed how tolerant the heart was of injury, providing certain portions of it were not injured. The walls of the heart were tolerant of injury so long as there was no hæmorrhage to stifle the heart's action. But there were cases of death immediately occurring from very small injuries to the nervous ganglia. —Mr. Frazer said that amongst the Japanese puncturing of the heart was a primitive mode of treatment for the cure of certain affections. He had a note of a *post-mortem* examination made while he was a student, in which he found a needle an inch and a half long in the surface of the heart, inside the pericardium, the surface of it being covered with old lymph.—Dr. Walter Smith mentioned a case of suicide which came within his knowledge. It was a young lady who became insane. One day, while two nurses were in the room with her, she drove a hair-pin, which she had secreted, into her abdomen through the umbilicus, uttering no sound whatever or expression of pain. She told the superintendent next day, that "she had done it." He looked at her abdomen, saw no mark, and disbelieved her; but symptoms of peritonitis soon appeared, and she died, and on a *post-mortem* examination the hair-pin was found amongst the coils of the intestine.—Mr. Molony mentioned a case of a person who attempted suicide by working a hole in the abdominal wall, about an inch on the left side of the umbilicus, with the end of a lucifer match. Mr. Cox gave a case of a man who, while suffering from delirium tremens in an asylum, wounded himself over the heart and in the arm with the sharpened end of a spoon.—*February Progress.*

